# Switching to boosted protease inhibitor plus a second antiretroviral drug (dual therapy) for treatment simplification: a multicenter observational study

**DOI:** 10.1186/s12879-016-1703-z

**Published:** 2016-08-11

**Authors:** Alessandra Latini, Massimiliano Fabbiani, Vanni Borghi, Gaetana Sterrantino, Alberto Giannetti, Patrizia Lorenzini, Laura Loiacono, Adriana Ammassari, Rita Bellagamba, Manuela Colafigli, Gabriella D’Ettorre, Simona Di Giambenedetto, Andrea Antinori, Mauro Zaccarelli

**Affiliations:** 1 San Gallicano Dermatologic Institute (IRCCS), Clinic of Dermatology and Infectious Diseases, Rome, Italy; 2 Department of Infectious Diseases, Catholic University of the Sacred Heart, Rome, Italy; 3 Division of Infectious Diseases, Department of Internal Medicine, San Gerardo Hospital, University of Milano-Bicocca, Monza, Italy; 4 Azienda Ospedaliero Universitaria, Clinic of Infectious Diseases, Modena, Italy; 5 Division of Infectious Diseases, Careggi Hospital, Florence, Italy; 6 Clinical Department, National Institute for the Infectious Diseases “Lazzaro Spallanzani”, Rome, Italy; 7 Policlinico Universitario “Umberto I”, Clinic of Infectious Diseases, Rome, Italy

**Keywords:** Antiretroviral therapy, Dual therapy, Treatment simplification, Darunavir Atazanavir, Raltegravir

## Abstract

**Background:**

Aim of the study was to assess predictors of discontinuation/toxicity of boosted PI-based (PI/r) dual therapy (DT).

**Methods:**

Observational, retrospective switch study in patients successfully treated with triple drugs regimen. Patients switched to PI/r based DT [darunavir (DRV/r), lopinavir (LPV/r) or atazanavir (ATV/r)] plus a second drug: [raltegravir (RAL), maraviroc (MVC) etravirine (ETR), lamivudine (3TC) or tenofovir (TDF)] between 2009 and 2014 were included. The effect of each drug as well as other clinical and virological cofactors over treatment discontinuation (TD) was assessed using survival analysis.

**Results:**

Overall, 376 patients were included with mean follow-up of 73 weeks. The most commonly used drugs in DT were DRV/r (63.0 %) and RAL (53.7 %). TD was observed in 77 (20,4 %) patients: 38 (10,1 %) virological failure, 35 (9,3 %) toxicity/intolerance (4 deaths) and 4 (1 %) interruptions for patients decision. At Cox Model, adjusted by demographic and laboratory variables, DRV/r and ATV/r significantly reduced the likelihood of TD and longer treatment was associated with lower risk, while low CD4 count at baseline and number of previous regimens with a higher risk. Moreover, RAL and 3TC use were significantly associated with lower TD by toxicity.

**Conclusions:**

In our clinical practice experience, switching virologically suppressed patients to PI/r based DT showed adequate safety and efficacy, so that it may be used in selected patients with specific medical needs.

## Background

The current standard combination antiretroviral therapy (cART) for HIV-1 infection restores the immune function by limiting the progression of the disease [1]. The introduction of cART in clinical practice significantly reduced HIV-related morbidity and mortality, improving the quality of life (QoL) of people infected with HIV [2]. An Italian National Cohort study showed that toxicity is the most frequent cause of discontinuation of cART [3]. In order to improve tolerance to new regimens as well as to increase adherence and durability of treatments, different strategies of simplification were investigated. Simplification of cART regimens and improved patient QoL today must also respond to cost/effectiveness criteria, making them affordable for local health systems.

The simplification strategies may include drug dose reduction and daily administration reduction (up to single tablet regimen, STR) or decrease in the number of antiretroviral drugs at time also defined as less drug regimen or LDR [4].

In particular, LDR strategies consist of dual therapy (DT) and mono-therapy (MT); in this setting, the most studied regimens include boosted protease inhibitors (PI/r) due to the high genetic barrier of this drug class. To date, the most relevant trials in the field are MT studies evaluating the role of boosted darunavir (DRV/r) MT [5, 6]. Both MT and DT strategies show generally disappointing results in naïve patients [7–12] with few exceptions for DT [11, 13], while encouraging results have been observed switching to DT or MT in patients with virological control of HIV infection both in clinical studies and real life setting [5, 6, 13–19].

Moreover, PI/r use in DT or MT regimens is associated to minimal or absent selection of drug-resistant HIV variants [20, 21]. LDR also offers the advantage of reduced toxicity associated with the use of nucleoside reverse transcriptase inhibitors (NRTI). Current literature provides adequate data from randomized clinical trials regarding the safety and efficacy of PI/r based DT in naïve patients [8–12, 22]. Recently, the first results from randomized clinical trials regarding DT in treatment simplification were presented [13, 14].

More data are required in order to better understand the real utility of simplification strategies and in particular of DT regimens in clinical practice. Thus, we aimed at assessing the durability of different DT regimens as cART simplification, in terms of treatment discontinuation or failure, and to evaluate the possible association of the different drug combinations to the outcome.

## Methods

### Study design and patients included

This is an observational and retrospective study from six Italian HIV reference centers enrolling patients on virologically effective HAART who, between 2009 and 2014, underwent DT regimen including PI/r and a second agent, according to clinician’s choice. Boosted PI were DRV/r, lopinavir (LPV/r) or atazanavir (ATV/r) and second agents were raltegravir (RAL), maraviroc (MVC), etravirine (ETR), lamivudine (3TC) or tenofovir (TDF). Patients were included if on successful HAART (HIV-RNA <50 copies/ml), no history of PI failure and no primary resistance mutations to PI or to second agents. Data were collected by investigators looking at any reason for starting DT regimen and in particular patients were enrolled if patients complained toxicity.

All patients of all the centers involved signed an informed consent for use of their clinical and laboratory data in aggregated and anonymous form and are aware that the databases can be used to produce observational studies. The procedure of collecting data was notified to the ethic committees of the centers. Since the study was performed within the data collected in the databases, no specific ethical committee approval was needed.

### Follow-up analysis

The follow-up was carried out up to DT discontinuation or to the last observation available if patients did not interrupted DT. During follow-up, virological and immunological data were recorded.

For patients who interrupted the treatment, date and reason of discontinuation were also recorded. Patients lost to follow-up, if in DT, were censored at the last follow-up visit. Discontinuation for cause as secondary endpoints (virological failure, toxicity or low adherence/patient’ decision) were also analyzed individually. Virological failure was defined as two consecutive detectable HIV-RNA (>50 copies/ml) or one detectable HIV-RNA at the last visit. Patients with two consecutive detectable HIV-RNA were considered as virological failure even if they continued DT.

In order to analyze the effect of the drugs used in DT, they were evaluated as predictors of the probability of discontinuation. Each drug was tested separately and, in the adjusted analysis, all drugs were included with all the other demographic, clinical and laboratory data in order to compare their effects.

### Statistical analysis

The statistical analysis was performed using the standard statistical methods for follow-up analysis: Kaplan-Meier survival analysis was used to assess probability of interruption; adjusted and unadjusted Cox proportional hazard model was used in order to evaluate the predictors of treatment interruption, including each of the drugs used in DT among the other variables.

## Results

### Descriptive results

Overall, 376 patients were included in the analysis.

The general characteristics of the patients are reported in Table 1. As shown, patients were generally strongly pre-treated both with a median long time in ART and a median high number of previous regimens by patient. DRV/r was the most used drug among PI/r and RAL among second drugs, so that the combination including DRV/r and RAL was the most frequently used (122 patients, 32.5 %). Among DRV/r treated patients, the dose of DRV used was available for 207 patients: 162 (72.9 %) were treated with 800 mg once daily (OD) and 56 (27.1 %) with 600 mg twice daily (BID).

**Table 1:** General characteristics of 376 patients with undetectable HIV-RNA switching to dual therapy

	N (%)	Median (IQ range)
Male gender	286 (76.1)	
Age		50 (45–55)
Non-Italian origin	38 (10.1)	
Years on cART		13 (7–16)
IDU as risk factor	98 (26.1)	
AIDS diagnosis	127 (33.8)	
N. of prev. regimens		5 (3–9)
CD4+ nadir		123 (46–243)
Baseline CD4+ count		534 (341–701)
HCV co-infection	123 (32.7)	
Prev. GRT for VF	238 (63.3)	
Switch to PI/r		
ATV/r	69 (18.4)	
DRV/r	237 (63.0)	
LPV/r	70 (18.6)	
Switch to second drug		
ETR	62 (16.5)	
RAL	202 (53.7)	
MVC	65 (17.3)	
3TC	23 (6.1)	
TDF	24 (6.4)	

*Abbreviations*: *cART* combined antiretroviral therapy, *IDU* injecting drug use, *HCV* hepatitis C virus, *GRT* genotypic resistance test, *VF* virological failure, *PI/rit* ritonavir-boosted protease inhibitor, *ATV/r* atazanavir/ritonavir, *DRV/r* darunavir/ritonavir, *LPV/r* lopinavir/ritonavir, *ETR* etravirine, *RAL* raltegravir, *MVC* maraviroc, *3TC* lamivudine, *TDF* tenofovir

In a median of 508 days of observations (interquartile range, IQR 198–995), 77 patients (20.5 %) discontinued DT; the reason for discontinuation was virological failure in 38 patients (10.1 %), toxicity in 35 (9.3 %), low adherence/personal decision in 12 (3.2 %). Reasons for DT discontinuation by toxicity included gastro-intestinal symptoms in 7 patients, lipid elevation in 6, non AIDS-related death in 4 (2 accidents, 2 non-AIDS related cancers), bilirubin elevation or liver toxicity in 4 patients each, kidney or CNS toxicity in 2 patients each, and it was unrecorded in 6.

The probability of discontinuation estimated by Kaplan-Meier survival analysis at one, three and five years is reported in Table 2. As shown, the probability of discontinuation was quite high in the first year of treatment, then increased during follow-up but at a slower rate. Discontinuation by virological failure was more frequent than by toxicity in long term observation.

**Table 2:** Estimated probability of dual therapy discontinuation by cause (Kaplan-Meier survival analysis)

	1 year	3 years	5 years
Overall	13.3 %	26.5 %	36.2 %
Virological failure	5.4 %	14.2 %	21.7 %
Toxicity	7.5 %	13.5 %	13.5 %

### Study drugs and overall risk of discontinuation

**Fig. 1:**
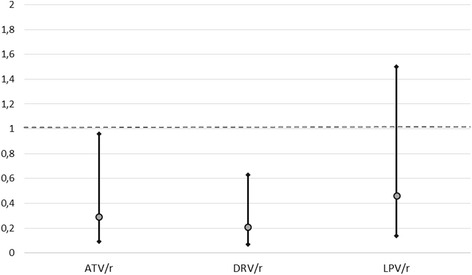
Adjusted HR at Cox Proportional Hazard Model for Dual-Therapy Discontinuation: PI/r

**Fig. 2:**
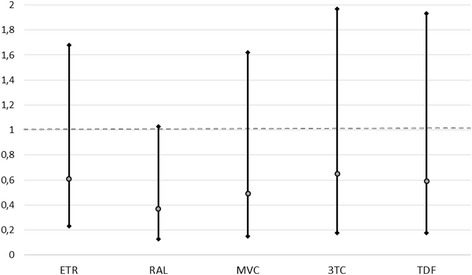
Adjusted HR at Cox Proportional Hazard Model for Dual-Therapy Discontinuation: Second Drug

Unadjusted and adjusted assessment for DT discontinuation using Cox proportional hazard model is reported in Table 3. As shown, at the unadjusted analysis, having less than 200 CD4 cells/μL at baseline and a higher number of previous regimens were significantly associated with a higher risk of treatment discontinuation; among drugs, only DRV/r use in DT was associated with a lower risk of discontinuation. In the adjusted model, among PI/s, ATV/r and DRV/r were associated with higher risk of discontinuation and, among second drugs, only RAL came close to a significant association with a lower risk of discontinuation (*p* = 0.056). Low CD4 cell count at baseline and higher number of previous regimens remained significantly associated to the outcome while a longer time on cART was associated with lower risk of discontinuation. The adjusted hazard ratio (HR) for DT discontinuation are also graphically shown in Fig. 1 (for PI/r) and Fig. 2 (for the second drug).

**Table 3:** Unadjusted and adjusted association with dual therapy discontinuation by any cause (Cox proportional Hazard Model)

	Unadjusted HR (95 % CI)	Adjusted HR (95 % CI)	*P* value
Male gender	1.08 (0.63–1.86)	1.12 (0.65–1.92)	n.s.
Age (by year)	1.09 (0.98–1.02)	1.00 (0.97–1.03)	n.s.
Non-Italian origin	0.45 (0.17–1.24)	0.43 (0.15–1.24)	n.s.
Years on cART (each)	0.97 (0.94–1.01)	0.94 (0.89–1.00)	0.033
IDU as risk factor	1.02 (0.93–1.12)	1.01 (0.91–1.11)	n.s.
AIDS diagnosis	1.19 (0.75–1.89)	0.84 (0.49–1.44)	n.s.
No. of previous regimens (per each one more)	1.04 (1.00–1.09)	1.10 (1.01–1.13)	0.027
CD4 nadir (by 50)	1.37 (0.84–2.21)	1.43 (0.81–2.52)	n.s.
<200 /mmc Baseline CD4 count	2.07 (1.19–3.60)	2.18 (1.18–4.03)	0.013
HCV coinfection	0.99 (0.61–1.60)	1.26 (0.75–2.11)	n.s.
Previous virological failure	0.89 (0.55–1.42)	1.14 (0.95–1.38)	n.s.
Switch to (PI/r)			
ATV/r	0.79 (0.41–1.49)	0.29 (0.09–0.99)	0.049
DRV/r	0.61 (0.39–0.96)	0.21 (0.07–0.63)	0.005
LPV/r	2.06 (1.25–3.42)	0.46 (0.14–1.50)	n.s.
Switch to (Second Drug)			
ETR	1.41 (0.83–2.40)	0.62 (0.23–1.68)	n.s.
RAL	0.67 (0.43–1.05)	0.37 (0.13–1.04)	n.s.
MVC	0.74 (0.34–1.62)	0.49 (0.15–1.62)	n.s.
3TC	1.66 (0.62–3.84)	0.65 (0.18–2.39)	n.s.
TDF	1.39 (0.67–2.91)	0.59 (0.18–1.93)	n.s.

In a further adjusted model who evaluated the effect of DRV/r at two different doses, both were significantly associated with reduced risk of discontinuation (HR 0.16, 95 % CI 0.05–0.58, *p* = 0.005 for 600 mg BID; HR 0.16, 95 % CI 0.05–0.47, *p* = 0.001 for 800 mg OD).

### Study drugs and risk of discontinuation by secondary end-points

None of the factors analyzed was significantly associated with DT discontinuation by virological failure in adjusted analysis; however, ATV/r (HR 0.17, 95 % CI 0.03–1.10, *p* = 0.065) and DRV/r use (HR 0.22, 95 % CI 0.05–1.12, *p* = 0.068) were marginally associated with lower risk of failure.

A lower risk of discontinuation due to toxicity was associated to RAL (HR 0.17, 95 % CI 0.04–0.67, *p* = 0.011) and 3TC use (HR 0.14, 95 % CI 0.02–1.00, *p* = 0.05) as well as to longer duration of cART (HR 0.93, 95 % CI 0.83–0.99, *p* = 0025).

Finally, DRV/r (HR 0.06, 95 % CI 0.01–0.79, *p* = 0.032), RAL (HR 0.09, 95 % CI 0.1–0.79, *p* = 0.029) and ETR use (HR 0.09, 95 % CI 0.01–0.89, *p* = 0.039) were associated with lower risk of discontinuation for inadequate adherence or patient decision.

Among 35 patients failing DT, 12 failed with HIV-RNA <1000 mc/ml, resistance at failure test was available for 18 patients (51.4 %) and, for 5 patients who failed RAL plus DRV/r regimen, also resistance test for integrase section of genome. One major mutation for protease (M46L) and one mutation for reverse transcriptase (M184V), probably due to re-emergence of NRTI resistance after failure, were detected. Both were found in two patients failing RAL plus DRV/r combination, while no integrase mutation was found.

## Discussion

The possibility to combine drugs with a high genetic barrier as PI/r to second drugs with different resistance profiles was demonstrated to be effective in pretreated HIV patients who failed other regimens [23, 24]. Favorable results were also observed in cART-experienced patients switched to DT after virological suppression with the aim of limiting short and long term NRTI-related toxicity. Not all PI/r were equally effective in DT regimens and their combination with different second drugs had various results in term of efficacy, safety and durability.

However, several studies have already shown not so favorable results of different DT regimens in first line: the MODERN study, which evaluated the association of DRV/r with MVC [10] and NEAT study [11], showed about 17 % of virologic failures as well as development of integrase resistance mutations in naïve patients with high baseline viral load failing first-line DT with DRV/r and RAL. Otherwise, the GARDEL study, also performed in first-line therapy, proved to be effective in naïve patients [22].

In contrast, induction-maintenance strategy, with simplification to a DT in patients with optimal virological response, showed more promising results [13, 16].

The present study offers the advantage of evaluating the results of cART simplification to DT in clinical practice with a long follow-up, since up to 80 % of patients remained in DT for a median of 73 weeks.

In our sample, DRV/r was the most frequently used PI/r, followed by LPV/r and ATV/r. Among the second drugs, RAL was the most commonly used followed by MVC and ETR. 3TC and TDF were used in few patients but significant differences in efficacy and safety were observed.

DRV/r in association with MCV, ETR or RAL showed positive results in recent pilot and cohort study [25, 26], while, the association of DRV/r with MVC showed disappointing results in randomized clinical trials [10, 27]. The use of DRV/r in combination with RAL was the most frequently DT used in our sample (one third of patients) as also observed in other non-randomized setting [24].

In our sample, RAL and 3TC as second drug showed the best safety profile and lower discontinuation rates, RAL is associated with lower toxicity, but in association with LPV/r showed less safe metabolic profile, in particular with the development of abnormal triglycerides level [16, 17].

In our analysis, the use of ATV/r and DRV/r were associated with a lower risk of discontinuation for virological failure, with low number of virological failure generally observed. Despite the efficacy of ATV/r used in MT or in combination with RAL in naïve and switched patients was shown to be disappointing [28–30], the use of the ATV/r has been shown to be non-inferior to triple therapy in randomized clinical trials, if associated with 3TC that offers better safety profile [13, 14].

DRV/r based DT was not associated with higher interruption by all causes neither combining nor distinguishing the two different doses of the drug in this study. Previous data demonstrated higher risk of virological failure associated with the use of DRV/r 800 OD vs 600 BID [26]. Currently, the DRV/r 800 OD schedule is considered as preferable and cost-effective [31].

Among second drugs, RAL has proven to be safer when compared with all other second drugs included in our analysis. Indeed, 20.5 % of discontinuation rate was observed in patients with low baseline CD4 cell count and this parameter was independently associated with a higher risk of discontinuation by all causes. In literature, RAL was associated to a high risk of virologic failure in case of lower baseline CD4 cell count [11] and of higher baseline viral load [8]. Finally, the use of ETR as second drugs could also be valid options in combination with PI/r, as already observed in several studies [15, 19].

Although the selection of patients included and data collection were accurate, our study may have several limitations. The study lacks of a control group as it was designed to compare the performance of single drugs used in DT. Moreover, the choice of including fully suppressed patients at DT initiation, excluding patients with previous virological failure to drugs used in DT, could overcome the absence of adherence data, not available in the study. Finally, data on changes of laboratory values are not presented as toxicity was evaluated if cause of discontinuation.

## Conclusions

Our analysis suggest that PI/r-based DT can be a useful switch strategy in HIV-infected patients with effective virological control overtime. Dual regimens should be used with caution in patients with low baseline CD4 cell count and high number of drugs in their history. Among PI/r, DRV appeared as the best option, followed by ATV/r, both associated with low probability of discontinuation; however, DRV/r appeared also to be the best tolerated. Among second drugs, RAL, due to its high tolerability, is related to the lowest risk of treatment discontinuation.

## Abbreviations

3TC, lamivudine; AIDS, Acquired Immuno-Deficiency Syndrome; ATV, atazanavir; BID, twice daily; cART, combination antiretroviral therapy; CNS, Central Nervous System; DRV, darunavir; DT, Dual Therapy; ETR, etravirine; HR, hazard ratio; IQR, interquartile range; LDR, less drug regimen; LPV, lopinavir; MT, mono-therapy; MVC, maraviroc; NRTI, nucleoside reverse transcriptase inhibitors; OD, once daily; PI, protease inhibitor; QoL, quality of life; r, ritonavir; RAL, raltegravir; STR, single tablet regimen; TD, treatment discontinuation; TDF, tenofovir; VL, virological failure
